# Talent management and job satisfaction of medical personnel in Polish healthcare entities

**DOI:** 10.3389/fpsyg.2023.1146073

**Published:** 2023-07-25

**Authors:** Wioletta Pomaranik, Magdalena Kludacz-Alessandri

**Affiliations:** College of Economics and Social Sciences, Warsaw University of Technology, Plock, Poland

**Keywords:** talent management, job satisfaction, medical personnel, healthcare entity, public sector

## Abstract

**Background:**

There is a mass exodus of qualified medical personnel in countries such as Poland. As a result, it is becoming increasingly important to study the satisfaction of medical personnel employed in public healthcare entities and the factors influencing this satisfaction. One such factor is the quality of talent management.

**Purpose:**

The study aimed to assess the quality of talent management in Polish healthcare entities and its impact on the job satisfaction of medical personnel. The study also considered the impact of other demographic, organizational and behavioral factors on medical personnel satisfaction, such as social competencies, job mobility, orientation toward the patient, gender and education stage.

**Methods:**

A questionnaire for healthcare professionals was used to collect data. A total of 747 respondents (506 defined as medical talent) participated in the survey. A 5-point Likert scale was adopted to assess job satisfaction and talent management practices. Reliability analysis was conducted to investigate the properties of this scale and the items that comprise it. The data was analyzed using descriptive statistics and structural equation modeling.

**Results:**

The survey showed that the quality of talent management in Polish healthcare institutions is not well assessed. Professional satisfaction of medical personnel working in Polish public healthcare entities depends mainly on talent management measured by talent motivation, talent development, employee appraisal and organizational culture. Among the factors that have a positive but smaller impact on job satisfaction are job mobility and the education stage. The impact of gender, patient orientation and social competence had the smallest but most significant impact on job satisfaction.

**Conclusion:**

Healthcare organizations should improve their talent management strategy to meet healthcare professionals’ current and future demands and improve their job satisfaction.

## 1. Introduction

One of the basic symptoms of the crisis in the Polish healthcare system is the limited availability of medical staff. Poland has one of the lowest ratios of the number of physicians and nurses *per capita* compared to other European Union countries (2.4 and 5.1 per 1,000 inhabitants, respectively) (OECD, 2020). At the same time, an increasing percentage of medical staff is in advanced age (over 60 years old), and the supply of young doctors and nurses is insufficient. Many years of acquiring and expanding professional qualifications, coupled with the inefficiency of the education system and post-graduate training, have resulted in shortages of specialized young medical personnel. In Poland, primary and specialist medical care systems require thorough changes and urgent action to improve the availability of medical services to the public through more effective human capital management (Leggat et al., 2020; Mitosis et al., 2021). The answer to the problems of human capital management in the Polish healthcare system may be an appropriate quality of the talent management system that can be used to anticipate and meet human capital needs in order to place the right people, in the right place, at the right time, to match the needs of medical staff to the needs and expectations of employers.

The field of talent management has gained considerable interest among researchers and practitioners over the past two decades (Barkhuizen and Gumede, 2021). This is because the best employees are the driving force behind an organization. It is on their imagination, charisma, skills and energy that the reputation of the organization in which they work depends to the highest degree (Mikuła and Morawski, 2009). Therefore, ensuring a quality supply of employee talent becomes essential. Modern management, especially the management of employees, seeks to make the fullest possible use of their potential. It is, therefore, important to identify their ambitions and, above all, their development opportunities, aptitudes and potential (Otola and Raczek, 2016).

The study focuses on four talent management practices (TMP): talent motivation, talent development, employee appraisal and organizational culture. This is due to the fact that talent management programs are not popular among healthcare entities in Poland. Talent management tools are simplified and focus mainly on these four practices. Little attention is paid to identifying talented employees, as managers of healthcare entities usually know which employees are key to the organization and focus on other practices to manage them better (Ingram and Glod, 2016).

Motivation is a complex and complicated process. This is due to the fact that each individual has different needs and goals (Paais and Pattiruhu, 2020). Based on research to date, it has been found that motivating factors in medical staff work, such as salary, the opportunity to improve skills through participation in courses and other forms of training, working conditions and cooperation in an interdisciplinary team, depending on the individual needs and situation of the healthcare entity. It should be noted that an important element of the motivation system is the recognition of employees who perform well. Fair remuneration is crucial for their retention in the organization. The study assumes that talent motivation in healthcare entities should be based on rewarding medical personnel for good performance and praising them, a fair remuneration system, and making employees aware that they work in a facility where the quality of medical services provided is taken care of.

Talent development is seen as a continuous process, the purpose of which is either to develop the capabilities and skills of the existing personnel of the organization in order to generate new talent or to develop the ability of recruited talented employees to occupy managerial positions (Mitosis et al., 2021). Talent development encompasses activities that help the best employees gain valuable skills and knowledge that can contribute to the organization’s development (Garavan et al., 2021). This practice includes activities that support the professional development of talent, such as training, coaching, and mentoring of high-potential medical personnel. The literature emphasizes that medical talent development is a key measure of talent management (Aljunaibi, 2014). It is essential for medical personnel to improve their skills in an ever-changing environment (Rabbi et al., 2015). The study assumes that the development of talent’s professional competencies in medical entities consists of the organization of in-house training, opportunities for staff to obtain career advancement, the allocation of financial resources for the development of medical talent, and the creation of educational programs for talents.

Talent appraisal is the process of evaluating people in an organization to identify high-potential employees. An organization should have a fair and acceptable appraisal system. Employee appraisal that takes place regularly allows verification of the correctness of the staff selection mechanisms in place, as well as the effectiveness of the training programs in place. Performance appraisal gives employees feedback on their work and behavior. Such an exercise can motivate employees to exert effort and behave appropriately in the workplace (Ismail et al., 2021). On the other hand, facility managers can use it to take corrective action or plan the professional development of medical personnel. The results of this appraisal also help with succession planning (Dzimbiri and Molefakgotla, 2021). Such assessment allows for the rational use of human capital potential and contributes to the better functioning of the organization as a whole (Bibi, 2019). The study assumes that employee appraisal in healthcare entities should occur periodically in the form of discussions that address the reasons for bad but also good employee performance. The way employees are evaluated should be transparent and objective. Managers are expected to provide feedback on the evaluation.

Building organizational culture is a talent management practice that is extremely exposed in the literature is building organizational culture (Bryan and Joyce, 2007; Górka et al., 2019). The organizational culture should strongly support talented employees, promote their distinctiveness and creativity and appreciate people who stand out. An important element in the organizational culture is trust and intra-organizational relations. These aspects facilitate the implementation of formal procedures, allow for honesty in behavior and develop teamwork. Organizational culture is a set of basic assumptions and beliefs of an organization’s employees developed to overcome external adaptation and internal integration problems (Limaj and Bernroider, 2019). Organizational culture consists of basic assumptions and beliefs shared by members of the organization that act unconsciously (Lund, 2003). Healthcare professionals’ perceptions of organizational culture describe the extent to which staff perceive the organization as having a set of expectations, guidelines, rules, and values that enable clear communication between members of the organization (Tzeng et al., 2002). As a key aspect of human resource management, talent management has a fundamental role in ensuring that employees are satisfied with their jobs so that they can stay in the organization.

In the literature, job satisfaction is a complex and multifaceted concept with many meanings for different researchers. Many authors understand job satisfaction as a person’s ability to realize needs, goals, beliefs and values (Locke, 1969). According to others, job satisfaction is the sum of people’s emotions, feelings, views and beliefs about their current job. Job satisfaction is an employee’s emotional state of fulfillment and achievement at work (Dzimbiri and Molefi, 2021). In other words, job satisfaction involves what employees enjoy doing, doing it perfectly, and being rewarded for their efforts. This means that job satisfaction is a key factor leading to a sense of accomplishment (Maurya and Agarwal, 2018). People’s job satisfaction levels can range from extreme satisfaction to extreme dissatisfaction. In addition to perceptions and attitudes about their job, employees may also have attitudes about various aspects of their career, such as the type of work they do, co-workers, supervisors or subordinates, and their salaries. Job satisfaction, therefore, includes employees’ state of mind and attitude in the workplace (Dzimbiri and Molefi, 2021). It is also an affective and cognitive assessment of what working conditions should be like and how they are perceived, which affects future expectations (Lu et al., 2005). Job satisfaction can be perceived by evaluating the workplace and its associated feelings (Kunecka, 2016). Thus, it should represent a balance between a person’s resources (education, commitment, experience) and what they gain in return (i.e., salary, promotion or relationships with colleagues). Job satisfaction is an emotional response in the form of feelings of pleasure related to the work performed or the performance of specific roles or functions. The concept of job satisfaction is often interchangeable with contentment as a synonym. However, the element that differentiates the two concepts is their timing. Contentment may be temporary, while an employee only experiences satisfaction after a prolonged period of contentment (Kunecka, 2016). Research shows that the feeling of contentment accompanying an employee influences their willingness to work and satisfaction with their work, which is consequently related to the quality of the service provided (Butkiewicz and Kaszkowiak, 2010). In summary, the level of job satisfaction reflects an individual’s level of fulfillment and positive and negative impressions during ongoing work.

The literature emphasizes that the quality of talent management influences employees’ job satisfaction (Barkhuizen and Gumede, 2021). Job satisfaction is essential because, in healthcare organizations, the mental state of employees influences patients’ satisfaction (Fernandes and Solimun, 2018). Satisfaction must therefore start with the service provider, which in this case is the employee of the medical entity. In addition, employee satisfaction is important because it contributes to the organization’s effectiveness (Jošanov-Vrgović and Pavlović, 2014). Keeping skilled employees happy reduces the likelihood of turnover, absenteeism and medical errors. In the international bibliography, several studies confirm the positive impact of various TMP on job satisfaction, which also positively affects the organization’s effectiveness (Aiken et al., 2015; De La Cruz and Abellan, 2015; Morais et al., 2017; Hendam et al., 2018; Albagawi, 2019; Alsubaie and Isouard, 2019; Kunecka and Skowron, 2019). Talent management strategies in healthcare that focus on staff satisfaction appear to positively impact many factors, including service quality, patient satisfaction, service efficiency, and organizational commitment. By implementing TM strategies to improve employee satisfaction, organizations can also acquire highly loyal employees to the organization’s values and goals.

In an attempt to link motivation to job satisfaction in healthcare workers, previous research has considered financial and non-financial motivators. Among the top motivators were achievements, salary, co-workers, and job characteristics (Lambrou et al., 2010). Important motivational factors that have been studied in connection with job satisfaction also included work atmosphere, management style, work environment, and recognition from superiors (Rouleau et al., 2012; Marinucci et al., 2013). The main factors positively influencing the satisfaction of healthcare workers were recognition from managers and co-workers and stable work/income. Low wages and difficult working conditions were the main factors that reduced job satisfaction. Intrinsic job satisfaction can be achieved when external needs, such as good working conditions or a satisfactory salary, have been satisfied, but also when the job is intrinsically satisfying, e.g., when it provides high social recognition or recognition of achievements by superiors or colleagues (Bonenberger et al., 2014). As the TMP influences employee job satisfaction, healthcare organizations can be effective by emphasizing talent development, which focuses on educating and training medical staff to develop their abilities, skills and knowledge. Recent studies examining the impact of all TM practices on job satisfaction concluded that talent development was the most important TMP influencing job satisfaction (Mabaso, 2020). These results align with previous studies’ results, which showed a significant relationship between talent development and job satisfaction (Haider et al., 2015; Chaudhary and Bhaskar, 2016). According to various researchers, units that provide their employees with training and development programs achieve higher employee satisfaction, retention and increased employee involvement in work (Chowdhury and Md. Nazmul H., 2017; Rezaei and Beyerlein, 2018; Paposa and Kumar, 2019; Almomani et al., 2022; Achmad et al., 2023). Training and development, mentoring, coaching and succession planning are tools for building a learning organization that can lead to employee satisfaction and greater organizational success (Rawashdeh, 2018). Employee appraisal can also be a talent management practice that positively affects job satisfaction (Sari Rosalina and Bancin, 2019; Subekti, 2021). This means that a better performance appraisal system will increase job satisfaction as it will become a benchmark for promotion as well as employee recognition. Employees’ satisfaction with the employee appraisal system used in the unit makes the employee happy to work. The existence of the appraisal system means that the employee knows his strengths and weaknesses better and can improve himself for the benefit of himself and the unit in which he works (Subekti and Setyadi, 2016). An appraisal system that provides opportunities for promotion can be the cause of productivity growth and can result in job satisfaction. On the other hand, an employee working under evaluation pressure cannot increase productivity (Akhter et al., 2016). Organizational culture influences employees’ thinking, solving problems, taking risks, and managing change. A strong culture is created when leaders and employees agree on core values, behaviors and the organization’s beliefs. An organization with a strong culture helps employees achieve their goals and tasks and be satisfied with their work (Tsai, 2011). Therefore, a pleasant working atmosphere incentivizes employees to achieve the organization’s goals. In contrast, employees’ dissatisfaction with the organization as a whole will affect their dissatisfaction in coping with their work (Ismail et al., 2021).

The literature emphasizes that other factors, such as job mobility, social competencies and patient orientation, can also influence job satisfaction (Hendam et al., 2018; Albagawi, 2019; Paais and Pattiruhu, 2020; Barkhuizen and Gumede, 2021).

The factor affecting job satisfaction may be the social competence of employees. Social competencies include communication and expressive skills, innovative and creative skills, collaborative skills, lifelong learning, problem-solving skills, positive attitudes and world views, interpersonal skills, critical thinking, and global views. They are considered essential competencies for effective job performance (Liu et al., 2019). Social skills are soft skills because they can influence others to get what they want and help individuals deal with critical issues that require multi-relationship cooperation. Medical personnel spend most of their time at work; work is central to staff identity, and attitudes toward work have key consequences (Lartey et al., 2019; Rubenstein et al., 2019). However, it should be noted that employees’ attitudes toward work are shaped by both workplace conditions and individual characteristics (Czajka, 1990). Social competence refers to engaging in meaningful interactions with others (Junge et al., 2020). For the purposes of the study, social competencies included: teamwork skills, commitment and responsibility, communication and relationship-building skills, time management effectiveness, assertiveness and the ability to work under stress and self-education.

Medical professions require a lot of commitment, and patients are increasingly seen as “customers” (Hudak et al., 2003). Therefore, it may be important for healthcare administration managers to understand employees’ orientation toward the patient. In a broader context, the patient-centered healthcare professional is a predisposition to care for patients’ needs (Skålén, 2010; Brown et al., 2018). Having customer orientation means genuinely wanting to attend to their needs and enjoying caring for them. This is a particularly important feature of medical staff who care for patients. This means that the desire to meet the patient’s needs is crucial to long-term happiness and success in the medical profession. Being “patient-centered” is fundamental to being satisfied and successful in healthcare (Wagner et al., 2012; Abugre et al., 2019). Customer orientation also regards individuals’ beliefs about their ability to meet the customer’s needs and the “pleasure” dimension, which assesses the degree to which customer interactions are inherently pleasant for service workers. Customer orientation is thus seen as a personality trait that is “critical to the service organization’s ability to orient itself to the market” (Brown et al., 2018).

The concept of job mobility can have more than one meaning. The concept may include moving between employers, between different types of contracts, between different professions and industries, or between different jobs. Geographical mobility can also be included in this concept, explaining changes in location and between countries (Berglund et al., 2010). For the purposes of this research, job mobility will be understood as an individual’s ability to move from one job to another and the ability to make sacrifices in order to find attractive employment (e.g., going abroad, changing the place of residence in the country, postponing plans to start or enlarge a family). The other researchers describe mobility as a willingness to voluntarily change jobs, which should not be confused with actual change (Lam et al., 2012). These days, employees are more likely to seek opportunities to change jobs to increase job satisfaction voluntarily. Therefore high job mobility can improve job satisfaction.

The study aimed to assess the quality of talent management in Polish healthcare entities and its impact on the job satisfaction of medical personnel. In order to identify TM factors influencing job satisfaction, the following dimensions were analyzed: talent motivation, talent development, employee appraisal and organizational culture. The study also considered the impact of other demographic, organizational and behavioral factors on medical personnel satisfaction, such as the social competencies possessed by the talent, job mobility, orientation toward the patient, gender and educational stage. Understanding the factors influencing the job satisfaction of medical personnel can contribute to improving the quality of work in public healthcare entities.

The article is structured as follows: in addition to this introduction, the following sections will discuss the research method and results. A discussion of the research findings will follow this. The end of the article features conclusions and recommendations.

The research contributed to gaining knowledge about the perceived quality of selected talent management practices and their relationship with the job satisfaction of medical staff. There is a need for scientific knowledge on the relationship between talent management and job satisfaction of medical staff in healthcare entities in Poland, as limited research in this area has been conducted in the context of Poland.

## 2. Methods

A survey questionnaire designed for medical personnel of Polish healthcare entities was used to collect the data needed for the analysis. A total of 747 respondents from across Poland took part in the survey. Based on the dimension “Sense of being talent,” a group of employees considering themselves as talents were identified. The variables used were: “I obtain above-average professional results,” “I have above-average talents in the medical direction,” “I have an aptitude for the medical profession,” “I have a strong influence on others” and “I consider myself talent for my future employer.” Cronbach’s alpha for this dimension takes a value of 0.704. As a criterion, more than half of the points possible within it (>13) were obtained from the indicator. This distribution formed a group of talented employees (*N* = 718). The medical talents employed in public healthcare entities were 506. The target analyses were conducted on this group.

The following socio-demographic characteristics were assessed in the study to determine the respondents’ description and whether they were well suited for the study: gender, age, educational stage and seniority in the profession (Table 1).

**Table 1:** Response by gender.

Gender (G)	Frequency (N)	Per cent (%)
Male	366	72.3
Female	140	27.7
Total	506	100.0

The study findings indicate that over half (72.3%) of the respondents in the study were female, and 27.7% were male. The findings therefore show that there was not relative gender balance among the staff of Polish healthcare entities as the number of female and male respondents was not close.

The age category of the respondents was also sought in this study (Table 2).

**Table 2:** Response by age.

Age category	Frequency (N)	Per cent (%)
Less than 30 years	56	11.1
Between 30–40 years	82	16.2
Between 41–50 years	120	23.7
Between 51–60 years	220	43.5
Above 60 years	28	5.5
Total	506	100.0

The study findings indicate that almost half (43.5%) of respondents were aged between 51 and 60. The mean age was 48 years. Therefore, most of the staff in Polish healthcare entities were not in their youth.

The study sought to establish the period under which the respondents worked with the healthcare entities. This was meant to establish whether the respondents could articulate the issues in this study relating to working in Polish healthcare entities. The results are shown in Table 3.

**Table 3:** Response by seniority in the profession.

Years of experience	Frequency (N)	Per cent (%)
Between 1–5 years	57	11.3
Between 6–10 years	35	6.9
Between 11–15 years	61	12.1
Between 16–25 years	85	16.8
Above 25 years	268	53.0
Total	506	100.0

The study findings indicate that the majority (53%) of the respondents in the study had a working experience of above 25 years, 16.8% had a working experience of between 16 to 25 years, 12.1% had a working experience of between 11 to 15 years, 11.3% had a working experience of between 1 to 5 years, 6.9% had a working experience of between 6 to 10 years. The fact that respondents had worked between 1 to 5 years and above illustrated that they were able to articulate the issues in this study.

The educational stage of the respondents was also sought in this study (Table 4).

**Table 4:** Response by educational stage.

Educational stages (ES)	Frequency (N)	Per cent (%)
Secondary education	40	7.9
Master’s degree	429	84.8
Doctoral degree and professor	37	7.3
Total	506	100.0

Most respondents had master’s degrees (85% of respondents). Medical staff with secondary education or doctoral and higher degrees each accounted for 7% of all respondents.

The questionnaire used in the study included variables to assess the job satisfaction of medical personnel (6 items) and four talent management practices: professional competence development (4 items), employee motivation (4 items), employee appraisal (4 items) and organizational culture (3 items). As a result of the analysis of the individual components of talent management in the surveyed facilities, it was possible to identify the following quality levels of sophistication of these practices: 5– very high, 4 – high, 3 – moderate, 2 – low, 1 – very low.

In addition, the following dimensions were included in the questionnaire: social competencies (6 items), job mobility (4 items), and patient orientation (2 items). Each response was rated on a 5-point Likert scale, from 1 (strongly disagree) to 5 (strongly agree). Participation in the study was voluntary. Confidentiality and anonymity were assured to the study participants. Reliability analysis was conducted to examine the properties of the measurement scale and the items that comprise it. The data was analyzed using descriptive statistics and structural equation modeling. In all tests, value of *p*s less than 0.05 were interpreted as statistically significant. Analyses were performed using the SPSS v. seven statistical packages, IBM AMOS v.27 and Microsoft Excel 365.

## 3. Results

### 3.1. Evaluation of talent management practices

For the purpose of assessing talent management practices in Polish healthcare entities, statements were prepared relating to four dimensions representing talent management practices: talent motivation (TM), talent development (TD), employee appraisal (EA) and organizational culture (OC).

In order to verify the constructs to analyze the quality of talent management practices, factor analysis was conducted using Principal Component Analysis, and Varimax rotation was used. The variables presented in Table 5 were used for the analysis. The model turned out to be adequate since all variables were sufficiently correlated and formed a reliable solution. The adequacy was confirmed by two tests: (OECD, 2020) the Kaiser–Meyer–Olkin test (KMO coefficient) (KMO = 0.918 > 0.8) and Bartlett’s significant sphericity test (*χ*^2^ = 4314.698, df = 105, *p* < 0.0001 < 0.05) and (Leggat et al., 2020) extracted communalities. The principal components method and Varimax rotation were used to identify significant factors. Extracted communalities, ideally, should be greater than 0.5. However, the 0.3 thresholds is accepted (Table 5).

**Table 5:** Extracted communalities during exploratory factor analysis.

Variable	Initial	Extraction
The facility where I work has a fair system of remuneration for employees.	1.000	0.543
I receive praise from my superiors for a job well done.	1.000	0.805
The opinion about the quality of the medical services provided by the organization influences my motivation to work.	1.000	0.521
Employees who perform well at work are rewarded.	1.000	0.749
The healthcare entity allocates funds for staff development.	1.000	0.772
Managers organize internal training.	1.000	0.640
My employer has learning and development programs to develop talent.	1.000	0.701
The facility offers opportunities for professional advancement.	1.000	0.686
The organization has a transparent and objective way of appraising staff.	1.000	0.787
Surveys to evaluate my work are carried out periodically.	1.000	0.819
The evaluation takes place in the form of a discussion, and the reasons for the employee’s bad, but also good, performance is addressed.	1.000	0.714
Managers provide feedback to staff on staff appraisals carried out.	1.000	0.752
The facility has a friendly atmosphere.	1.000	0.754
The conditions of employment at the facility allow for a work-life balance.	1.000	0.698
Managers create a professional atmosphere in which respect is shown to medical personnel.	1.000	0.710

All variables in the model extracted communalities greater than 0.5. The model explains almost 71.01% of the variance. Four latent variables were expected to be extracted (Table 6).

**Table 6:** Rotated component matrix.^*^

Variable	Component			
	1 (TA)	2 (TD)	3 (OC)	4 (TM)
Surveys to evaluate my work are carried out periodically.	0.863			
The organization has a transparent and objective way of appraising staff.	0.776			
The evaluation takes place in the form of a discussion, and the reasons for the employee’s bad, but also good, performance is addressed.	0.756			
Managers provide feedback to staff on staff appraisals carried out.	0.655			
The healthcare entity allocates funds for staff development.		0.783		
The facility offers opportunities for professional advancement.		0.766		
My employer has learning and development programs to develop talent.		0.756		
Managers organize internal training.		0.679		
The facility has a friendly atmosphere.			0.836	
The conditions of employment at the facility allow for a work-life balance.			0.778	
Managers create a professional atmosphere in which respect is shown to medical personnel.			0.665	
I receive praise from my superiors for a job well done.				0.836
Employees who perform well at work are rewarded.				0.665
The opinion about the quality of the medical services provided by the organization influences my motivation to work.				0.529
The facility where I work has a fair system of remuneration for employees.				0.526

^*^Extraction Method: Principal Component Analysis, Rotation Method: Varimax with Kaiser Normalization.

The convergent validity of the model was confirmed. All variables’ loadings were greater than 0.5, and the average loading per latent factor was greater than 0.7 (Table 6), which means that each latent factor variable was highly correlated. None of the initial variables were removed. Additionally, discriminant validity was confirmed. All latent factors are unique and discriminant from each other. Variables load clearly on latent factors. There were no cross-loadings.

The responses of physicians, nurses, midwives and others surveyed and the Cronbach’s Alpha coefficient value for each dimension are presented in Table 7.

**Table 7:** Descriptive statistics for dimensions of talent management.

Construct and Cronbach’s alpha	Variables	Mean	Std.dDeviation
Talent Motivation (TM) *α* = 0.782	TM1. The facility where I work has a fair system of remuneration for employees	3.36	1.488
	TM2. I receive praise from my superiors for a job well done	3.69	1.469
	TM3. The opinion about the quality of the medical services provided by the organization influences my motivation to work	4.21	1.174
	TM4. Employees who perform well at work are rewarded	3.05	1.536
Talent development (TD) *α* = 0.836	TD1. The healthcare entity allocates funds for staff development	3.44	1.500
	TD2. Managers organize internal training	4.10	1.272
	TD3. My employer has learning and development programs to develop talent	2.91	1.524
	TD4. The facility offers opportunities for professional advancement	3.53	1.435
Employee appraisal (EA) *α* = 0.873	EA1. The organization has a transparent and objective way of appraising staff.	3.88	1.359
	EA2. Surveys to evaluate my work are carried out periodically	3.79	1.443
	EA3. The evaluation takes place in the form of a discussion, and the reasons for the employee’s bad, but also good, performance is addressed.	3.15	1.616
	EA4. Managers provide feedback to staff on staff appraisals carried out	3.34	1.611
Organizational culture (OC) *α* = 0.789	OC1. The facility has a friendly atmosphere	4.03	1.125
	OC2. The conditions of employment at the facility allow for a work-life balance	3.77	1.336
	OC3. Managers create a professional atmosphere in which respect is shown to medical personnel	3.79	1.386

The reliability of the model was confirmed. The high reliability of the scale is indicated by values of this coefficient greater than 0.7 (Rezaei and Beyerlein, 2018).

Respondents rated rewarding good performance as the lowest of all the variables describing the dimension of employee motivation (TM4: *M* = 3.05; SD = 1.536). This statement was completely disagreed with by 28.9% of the respondents and partially disagreed with by 9.1%. The study shows that talented medical personnel feel undervalued. Approximately 57.3% of the respondents believe that the opinion of the quality of the medical services provided by the facility where they work influences their motivation to work (TM3: *M* = 4.21; SD = 1.174). 24.5% of the respondents partially agree with this statement. The result makes this aspect the highest rated in the dimension concerning the motivation of medical personnel in public healthcare entities. The medical talents also commented on whether there is a fair system of employee remuneration in the facility where they work (TM1: *M* = 3.36; SD = 1.488). Approximately 34% of the medical talents surveyed completely or partially disagreed with this statement. Respondents also responded to the statement regarding receiving praise from a superior for a job well done (TM2: *M* = 3.69; SD = 1.469). About 40% of the respondents agreed with this statement.

The second dimension covered the development of professional competencies of employees. Of all the variables describing the development of competencies in public health care entities, respondents gave the best rating to the organization of internal training by managers (TD2: *M* = 4.10; SD = 1.272). The survey showed that most respondents (87.2%) completely or partially agreed with this statement. Medical talents also responded to the statement regarding the possibility of career advancement within the public medical entity where they work (TD4: *M* = 3.53; SD = 1.435). Approximately 16% of respondents completely negated this statement. Of all the variables describing the dimension of competence development, the respondents rated the public health care entity’s possession of training programs that aim at talent development the lowest (TD3: *M* = 2.91; SD = 1.524). Thirty percent of the respondents completely disagreed with this statement, and 11.7% partially disagreed. Approximately 19% of the respondents commented that, in their opinion, healthcare entities do not allocate financial resources for staff development (TD1: *M* = 3.44; SD = 1.500).

The third dimension concerns employee appraisal. According to 47.2% of the medical talents surveyed, there is a transparent and objective way of employee appraisal in the facility where they work (EA1: *M* = 3.88; SD = 1.359). The survey showed that most respondents (65.4%) completely or partially agreed with the statement that in the public medical entities where they work, employee appraisal occurs cyclically (EA2: *M* = 3.79; SD = 1.443). Respondents were also asked whether they received feedback on their employee appraisal (EA4: *M* = 3.34; SD = 1.610). 21.9% of respondents completely disagreed with this statement, while 14% partially agreed. Of all the variables describing the dimension of employee appraisal, the respondents gave the lowest rating to the form of appraisal (EA3: *M* = 3.15; SD = 1.616). The statement that in the organization where they work, employee appraisal takes place in the form of a discussion during which the reasons for bad as well as good employee performance are discussed was completely disagreed with by 25.7% of the medical talents surveyed.

The highest-rated variable among the organizational culture dimension by the medical talents concerned the friendly atmosphere in the public healthcare provider with which they had an employment relationship (OC1: *M* = 4.03; SD = 1.125). Aspects including a work-life balance (OC2: *M* = 3.77; SD = 1.336) and a professional working atmosphere in which medical personnel are shown respect (OC3: *M* = 3.79; SD = 1.386) fared slightly worse.

In interpreting the mean score rating in determining the level of talent management, the scale was as follows (Table 8).

**Table 8:** The quality of talent management (Yusof et al., 2015; Bongcayat and Guhao, 2020).

Mean score range	Descriptive quality	Interpretation
4.20–5.00	Very High	This indicates that talent management practice is always observed.
3.66–4.19	High	This indicates that talent management practice is frequently observed.
2.33–3.65	Moderate	This indicates that talent management practice is sometimes observed.
1.80–2.32	Low	This indicates that talent management practice is rarely observed.
1.00–1.79	Very Low	This indicates that talent management practice is never observed

The averages for the various dimensions of talent management in public healthcare entities in the medical talent group are as follows:

- *M* = 3.58 for employee motivation;
- *M* = 3.50 for the development of employees’ professional competencies;
- *M* = 3.54 for employee appraisal;
- *M* = 3.86 for organizational culture.

When interpreting the data, it should be noted that none of the dimensions of talent management in public healthcare entities (in addition to organizational culture), in the opinion of medical talent, was classified as high quality. According to medical talent, the biggest challenge for managers of public facilities is to ensure the development of employees’ professional competencies.

### 3.2. Evaluation of job satisfaction of medical staff

Six statements (S1, S2, S3, S4, S5, S6) were assigned to this dimension in the developed survey questionnaire to assess the job satisfaction of medical employees. The statements were responded to by 506 gifted medical employees of public healthcare entities (representing 100% of the results below). The mean values of the job satisfaction (JS) dimension ranged from 2.64 to 4.60. The measures of the correctness of the representation of the JS dimension were correct, so it was assumed that the variables correctly represent the JS dimension. Kaiser–Meyer–Olkin Measure of Sampling Adequacy: KMO = 0.774 > 0.6; Bartlett’s Test of Sphericity: χ^2^ = 692.181; DF = 15, *p* − value<0.001 (Tabachnick and Fidell, 2018). In this case, the reliability of the scale was examined based on Cronbach’s Alpha coefficient, which indicates whether the way the questions were answered was consistent. The high reliability of the scale is indicated by a Cronbach’s Alpha value greater than 0.7 (Rezaei and Beyerlein, 2018) (Table 9).

**Table 9:** Descriptive statistics for the job satisfaction dimension (*N* = 506).

Construct and Cronbach’s alpha	Variables	Mean	Std. deviation
Job satisfaction (JS) α =0.715	JS1. I identify with my current employer	4.32	1.165
	JS2. I have good contact with my superiors	4.35	1.052
	JS3. I have good contact with colleagues	4.60	0.572
	JS4. The amount of my current salary is satisfactory	3.46	1.406
	JS5. I do not feel an excessive workload	2.64	1.454
	JS6. I do not experience work burnout	3.61	1.444

**Figure 1:**
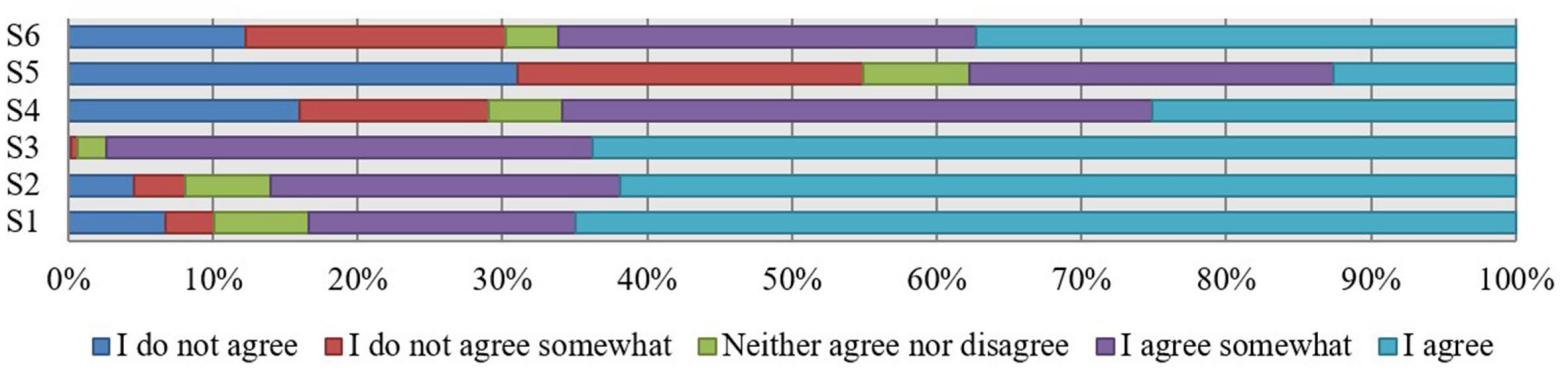
Satisfaction with work in public healthcare entities of surveyed medical talents (in %).

Cronbach’s alpha for this dimension takes a value of 0.715. The responses of doctors, nurses, midwives and others surveyed, expressing their satisfaction with their work in a public health facility, are shown in Figure 1.

The survey shows that 65% of respondents identify with their current employer (JS1). The following disagreed with this statement: completely 6.7% of the respondents and partly 3.4% (*M* = 4.32; SD = 1.165). Thus, about 10.1% will not identify with the organization where they work.

Those considering themselves to be medically talented responded to the statement that they have good contact with their superiors (JS2: *M* = 4.35; SD = 1.052). This statement was completely confirmed by 61.9% of respondents and partially confirmed by 24.1% of respondents. 4.5% of respondents are completely dissatisfied with their relationship with their superior, and 3.6% of respondents are partially dissatisfied.

The majority of medical talents (63.8%) report that they have good relationships with their colleagues. This statement was partially agreed with by 33.6% of respondents. Those unable to define their contact with other medical personnel and those describing these relationships as bad represent altogether 2.6% of all medical talents employed in public healthcare entities (JS3: *M* = 4.60; SD = 0.572).

Approximately 16% of the talents surveyed believe their salary is unsatisfactory. The opposite view is represented by 25.1% of the respondents. The largest group (40.7%) says they are rather satisfied with their salary (JS4: *M* = 3.46; SD = 1.406).

The majority of medical talents completely (31%) or partially (23.9%) agreed with the statement that they felt their workload was excessive (JS5: *M* = 2.64; SD = 1.454). About 12.6% of the respondents answered that their workload was adequate.

Professional burnout was felt by 12.3% of the medical talents surveyed (JS6: *M* = 3.61; SD = 1.444). In contrast, 18% of those working in public healthcare entities partially agreed with this statement. Some 37.4% of the respondents firmly answered that this problem does not concern them.

The averages for the individual medical personnel job satisfaction variables are in the range: 2.64 < *M* < 4.60. Interpreting the data, it should be noted that respondents were most satisfied with contact with colleagues (*M* = 4.60). In the opinion of medical talents, the rational workload of medical personnel is the biggest challenge for managers of Polish healthcare institutions (*M* = 2.64).

### 3.3. Analysis of the other dimensions covered by the survey

Talented medical personnel of public healthcare entities were also asked for their opinions on social competencies, patient orientation and job mobility (Table 10).

**Table 10:** Descriptive statistics of survey variables (*N* = 506).

Construct and Cronbach’s alpha	Variables	Mean	Std. deviation
Patient orientation (PO) α = 0.694	PO1. I care about patients and try to understand their points of view	4.71	0.568
	PO2. I seek to build a long-term relationship with the patient based on trust	4.49	0.776
Job mobility (JM) *α* = 0.725	JM1. For the sake of gaining attractive employment, I am able to go abroad	1.59	1.119
	JM2. For the sake of gaining attractive employment, I am able to change my place of residence in the country	1.84	1.323
	JM3. For the sake of gaining attractive employment, I am able to postpone plans for starting/expanding a family	1.74	1.140
	JM4. For the sake of gaining attractive employment, I am able to accept long commutes to work	2.08	1.432
Social competence (SC) *α* = 0.759	SC1. I can work in a team	4.63	0.870
	SC2. I manage my time effectively	4.33	0.963
	SC3. Self-education is my strength	4.19	1.025
	SC4. I have communication and social relationship-building skills	4.46	0.956
	SC5. I am an assertive person and able to work under stress	4.28	1.062
	SC6. I demonstrate commitment and responsibility	4.78	0.606

The study shows that gifted medical personnel rate the social competencies they possess very highly. The following deserve special mention: ability to work in a team (SC1: *M* = 4.63; SD = 0.870), commitment and responsibility (SC6: *M* = 4.78; SD = 0.606), ability to communicate and build relationships (SC4: *M* = 4.46; SD = 0.956). Respondents rated their own time management effectiveness (SC2: *M* = 4.33; SD = 0.963), assertiveness and ability to work under stress (SC5: *M* = 4.28; SD = 1.061) and self-education (SC3: *M* = 4.19; SD = 1.025) slightly lower.

Medical talents were also asked to indicate their level of patient orientation. Caring about patients and understanding their points of view was the best-rated aspect of this dimension (PO1: *M* = 4.71; SD = 0.568). The second component concerned building a long-term relationship with the patient based on trust (PO2: *M* = 4.49; SD = 0.776). Mean values of the patient orientation (PO) dimension ranged from 4.49 to 4.71, and the dimension was consistent. Cronbach′s − Alpha = 0.694 > 0.6.

Mean values of the job mobility (JM) dimension ranged from 1.59 to 2.08, and the dimension was consistent. Based on reliability analysis and factor analysis, it was assessed that statements JM1 to JM4 describing the JM dimension measure it correctly. Cronbach’s – Alpha = 0.725 > 0.6; Kaiser–Meyer–Olkin Measure of Sampling Adequacy: KMO = 0.664 > 0.6; Bartlett’s Test of Sphericity: *χ*^2^ = 405.920; DF = 6, *p* − value<0.001. The predicted utility measures of all variables were determined by imputing the responses from each dimension variable after conducting Confirmatory Factor Analysis (Wang B. et al., 2020). The talented medical professionals surveyed rated their job mobility very low. In order to get a more attractive job than their current one, they would not be able to: go abroad (JM1: *M* = 1.59; SD = 1.119), postpone plans to start or expand a family (JM3: *M* = 1.74; SD = 1.140), change their place of residence in the country (JM2: *M* = 1.84; SD = 1.323). The long commute was a slightly more acceptable inconvenience (JM4: *M* = 2.08; SD = 1.432). The study thus shows that talented medical staff are unwilling to make personal sacrifices to gain more attractive employment. For managers of public healthcare entities, this is very valuable information.

### 3.4. Confirmatory factor analysis

The whole model is also consistent. Measure of Sampling Adequacy: KMO = 0.875 > 0.6; Bartlett’s Test of Sphericity: χ^2^ = 9524.565; DF = 528, *p* − value<0.0001. The model explains 67.50% of data variance. Factor extraction results are presented in Table 11.

**Table 11:** Total variance explained by the model^*^.

Component	Initial eigenvalues			Extraction sums of squared loadings			Rotation sums of squared loadings		
	Total	% of Variance	% Cumulative	Total	% of Variance	% Cumulative	Total	% of Variance	% Cumulative
1	9.598	29.086	29.086	9.598	29.086	29.086	4.439	13.452	13.452
2	4.573	13.859	42.945	4.573	13.859	42.945	4.026	12.199	25.651
3	1.710	5.182	48.127	1.710	5.182	48.127	3.634	11.013	36.664
4	1.576	4.776	52.903	1.576	4.776	52.903	3.157	9.567	46.231
5	1.512	4.583	57.486	1.512	4.583	57.486	2.279	6.907	53.137
6	1.301	3.944	61.429	1.301	3.944	61.429	1.742	5.279	58.416
7	1.025	3.107	64.537	1.025	3.107	64.537	1.644	4.982	63.398
8	0.978	2.962	67.499	0.978	2.962	67.499	1.353	4.101	67.499
9	0.914	2.770	70.269						
10	0.867	2.628	72.897						
11	0.787	2.384	75.281						
12	0.708	2.145	77.425						
13	0.695	2.107	79.532						
14	0.621	1.882	81.414						
15	0.568	1.722	83.136						
16	0.526	1.595	84.731						
17	0.484	1.466	86.198						
18	0.453	1.372	87.570						
19	0.436	1.320	88.889						
20	0.432	1.308	90.198						
21	0.372	1.128	91.326						
22	0.349	1.058	92.384						
23	0.328	0.994	93.379						
24	0.320	0.969	94.348						
25	0.292	0.885	95.232						
26	0.280	0.849	96.081						
27	0.252	0.765	96.846						
28	0.219	0.663	97.509						
29	0.207	0.627	98.135						
30	0.175	0.529	98.664						
31	0.169	0.511	99.175						
32	0.146	0.441	99.617						
33	0.127	0.383	100.000						

^*^Extraction Method: Principal Component Analysis.

The relationship among the variables is checked through correlation analysis to determine whether the variables are highly correlated (Masood et al., 2020). The findings are shown in following Table 12.

**Table 12:** Cross-correlations matrix.

Correlations	JS	TMP	PO	JM	G	ES	SC
JS	1						
TMP	0.755*	1					
PO	0.285*	0.259*	1				
JM	0.403*	0.438*	0.099*	1			
G	−0.133*	−0.163*	−0.063	−0.112*	1		
ES	−0.183*	−0.159*	−0.054	−0.053	0.298*	1	
SC	0.273*	0.193*	0.271*	0.173*	−0.357*	0.035	1

^*^Refers to *p* < 0.05.

Using a structural model, regression analysis was performed in IBM SPSS AMOS v. 27.0 software. The following null hypotheses were developed to guide the research:

Null Hypotheses 1: TMP does not affect JS.

Null Hypotheses 2: JM does not affect JS.

Null Hypotheses 3: PO does not affect JS.

Null Hypotheses 4: G does not affect JS.

Null Hypotheses 5: ES does not affect JS.

Null Hypotheses 6: SC does not affect JS.

**Figure 2:**
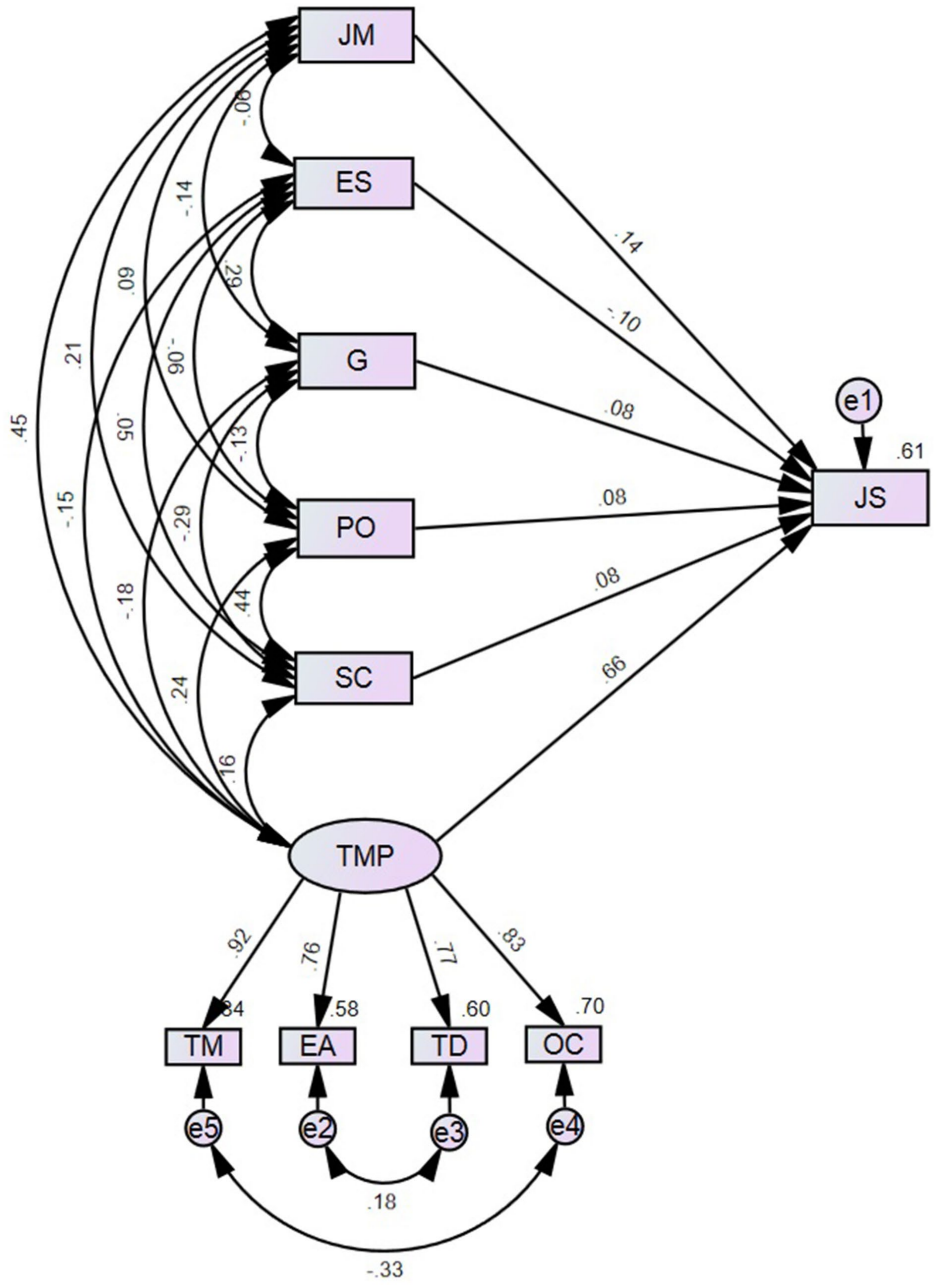
The structural model with the determined standard path loadings.

Hypothesis testing is done by analyzing the significance of path coefficients. If the path coefficient’s value of *p* is ≤0.05 (5%), the null hypothesis can be rejected, meaning that independent variables significantly affect the dependent variable. Figure 2 shows the structural model with the determined standard path loadings.

The values of the model evaluation coefficients, CFI = 0.938 (>0.8), GFI = 0.978 (>0.95), AGFI = 0.932 (>0.90), C_min/DF = 3.073 (between 1 and 5), RMSEA = 0.064 (<0.1), *p*-close = 0.103 (>0.05) confirmed the reliability of the model. The model was considered correct regarding the requirements for structural models (Tabachnick and Fidell, 2018).

Standardized and unstandardized path loadings are presented in Table 13.

**Table 13:** Standardized and unstandardized model path loading.

Hypothesis	Path	Unstandardized estimate	Standardized estimate	S.E.	C.R.	Value of *p*	Hypothesis acceptance
H1	TM to JS	0.549	0.663	0.040	13.884	<0.0001	Accepted
H2	JM to JS	0.085	0.137	0.024	3.578	<0.0001	Accepted
H3	PO to SJ	0.152	0.080	0.070	2.180	0.029	Accepted
H4	ES to SJ	−0.574	−0.100	0.168	−3.407	<0.0001	Accepted
H5	G to SJ	0.430	0.085	0.176	2.450	0.014	Accepted
H6	SC to SJ	0.045	0.078	0.023	1.964	0.050	Accepted

S.E., Standard Error; C.R., Critical Ratio; *P*, *p*-value.

Hypotheses H1, H2, H3, H4, H5, and H6 were confirmed. Talent management (TMP) has the highest impact on job satisfaction (JS). The impact of job mobility (JM) and education stage (ES) are smaller. The smallest but most significant impact on JS had gender (G), patient orientation (PO) and social competence (SC).

## 4. Discussion

The research showed that talent management practices are currently poorly assessed by healthcare personnel of healthcare entities in Poland. This may be due to the fact that these medical entities implement various strategies for developing, motivating and evaluating talented medical employees to a small extent. The literature emphasizes that a high quality of talent management practices is achieved when they focus on a specific group of healthcare professionals in the face of a specific phenomenon (usually a shortage of specialists) (Thompson and Ahrens, 2015; Yi et al., 2015). Talent management in healthcare has been rated as low level also by medical staff from other countries where research has been conducted in this area, for instance, by nurses in public hospitals in Malawi (Dzimbiri and Molefakgotla, 2021). The results showed that almost 70% of respondents rated their talent management practices as poor or very poor. Poor employment practices have also been observed in Nigeria (Lawal et al., 2022). The average TMP quality was observed among employees in private hospitals in Libya (Alferjany et al., 2022). However, it is worth emphasizing that different TM practices were assessed in other countries, and therefore, comparing these results should be approached with caution. Poor assessment of talent management was also obtained in research conducted among midwives working in hospitals in Iran (Kheirkhah et al., 2016).

In our study, the practices of organizational culture and talent motivation were rated the best and the practice of professional development the worst. Other studies conducted in this area revealed that talent development practice is rated the highest in public sector organizations (Yener et al., 2017; Mahfoozi et al., 2018). For instance, this TM practice received the highest scores in studies conducted among midwives in Iran (Kheirkhah et al., 2016). This practice is well perceived when employees’ aspirations coincide with the organization’s needs (Kadam et al., 2016). In this situation, talent management focuses on training through a cognitive approach emphasizing competency-based development (Subramaniam et al., 2015). The situation in the Polish healthcare sector is different, as the needs of employees who are often overloaded with work do not coincide with those of medical entities that need more medical staff (Fiałek, 2022). In our study, the best-rated TM practices turned out to be organizational culture and talent motivation. By contrast, in other studies, these practices were the worst-assessed TMP (Groves, 2018).

Job satisfaction of medical employees in Poland was assessed at quite a high level. Aspects that lower the level of satisfaction include excessive workload (2.64) and occupational burnout (3.61). It is worth emphasizing that job satisfaction is usually rated low in the field of healthcare around the world (Mouhamadou et al., 2017). Low satisfaction factors in other countries include high levels of burnout, long working hours, mobbing in the workplace, demanding jobs, insufficient management support, time-consuming regulatory obligations and many other factors (Adriaenssens et al., 2015; Allen et al., 2015; Jesse et al., 2015; Cao et al., 2016). Our research found that talent management is the most important factor positively impacting employee job satisfaction. Our study covers four practices: talent motivation, talent development, employee appraisal, and organizational culture. This is supported by other research showing that such talent management practices affect employee satisfaction (Bhatnagar, 2007; Ingram, 2016; Sinclair-Maragh et al., 2017; Baroda, 2018; Schreuder and Noorman, 2019).

According to this research, employees will feel valued if the organization takes care of them and gives them opportunities for professional development and training or if the organization has a fair and acceptable appraisal system. According to Hans (2015), this can create a pleasant working atmosphere in which employees are motivated to achieve the organization’s goals. Also, other researchers indicated that remuneration-based motivation, training and support in professional development were considered to be key issues of satisfaction of healthcare workers (Anlesinya et al., 2019). A study conducted in India found that all talent management practices in healthcare organizations can increase nurses’ job satisfaction and organizational commitment (Gül et al., 2022).

Talent motivation is of key importance in the practice of talent management in Polish healthcare entities (0.91). In our study, the assessment of this practice included the system of compensation for employees, rewards, opinions about the quality of the medical services and praise from superiors. Other studies confirming the positive impact of motivation on employee satisfaction also found that paying more attention to payroll, employee recruitment benefits, rewards and additional payments is important to motivate talent and strengthen their loyalty to the organization (Ben Matug, 2019). Elements of talent motivation that have been noted to affect job satisfaction include payment (Lum et al., 1998), performance rewards, relationships with superiors and other benefits (Wilson, 2006). From a motivational point of view, if extrinsic rewards are valued by medical staff, they will contribute to better work effort, job satisfaction, and retention (Harris et al., 2008).

Excessive workload, unsatisfactory salaries and stress in the professional environment result in professional burnout (Binczycka-Anholcer and Lepiesza, 2011; Nowakowska, 2016). In the Polish healthcare sector, employees are overloaded with work (2.64) and complain about professional burnout (3.61). The lower satisfaction of medical personnel in public healthcare entities can also be due to their unsatisfactory remuneration (3.46). Therefore, the management of healthcare entities in Poland must recognize and influence the key role of effective talent motivation in improving job satisfaction. Most healthcare studies to date point to a single impact of organizational culture on job satisfaction (Tzeng et al., 2002; Lund, 2003). Organizational culture is often considered a prerequisite for teamwork in an organization. This means that establishing good interprofessional cooperation in the team is the most important thing in order to increase job satisfaction in healthcare (Körner et al., 2015). In line with previous studies (Bleakley et al., 2009; Körner, 2010; O’Leary et al., 2012; Brennan et al., 2013), this highlights the importance of high-quality cross-professional teamwork in healthcare. Recent studies of medical practices that have shown that organizational culture is related to physician job satisfaction also underlined that a stronger evidence base is required in this area (Zazzali et al., 2007; Brazil et al., 2010). Only Research in Indonesia has shown that organizational culture does not significantly affect employees’ job satisfaction (Paais and Pattiruhu, 2020). Also, a study conducted among hospital administrative employees showed that the impact of organizational culture on job satisfaction is not significant (Dimitrios et al., 2014).

Our study showed that medical personnel in Poland have an above-average level of social competence. In turn, studies conducted in other countries have shown the problem of insufficient soft skills in the group of doctors, students and graduates of medical universities (Brown and Crookes, 2016; Missen et al., 2016; Kasam et al., 2020; Roller and Eberhard, 2021). Respondents rated the highest the commitment and responsibility (4.78), the ability to work in a team (4.63) and the ability to communicate and build social relations (4.46). In the literature on the subject, special attention is paid to such competencies as creativity, dedication, teamwork, skills, interpersonal relations and worldview (Liu et al., 2019). They are believed to enable individuals to be integrated into society.

Our study shows a significant impact of social competencies on the job satisfaction of medical staff. Therefore, previous research showing that well-developed social competencies can improve professional skills, reduce stress and increase job satisfaction has been confirmed (Bernburg et al., 2016). It is worth emphasizing, however, that these studies focused mainly on one element of job satisfaction: the control of occupational burnout (Tarcan et al., 2017). It has been shown that a high level of social competence is a key element in improving satisfaction with medical practice among health professionals at risk of professional burnout (Kurunsaari et al., 2018). Therefore, it is important to combine clinical and social competencies in the medical profession, as it supports the implementation of patient-centered care, and social competencies protect medical staff against stress and professional burnout (Haley et al., 2017; Losa-Iglesias et al., 2018; Chrzan-Rodak et al., 2022).

Respondents in this study were characterized by a very low degree of professional mobility. In addition, the analysis results showed that professional mobility significantly positively influenced job satisfaction, which was confirmed by previous research (Acheampong et al., 2010). From an individual point of view, mobility is an investment in human capital, thanks to which some workers are able to increase their earnings, as well as job satisfaction and personal well-being (Nakosteen et al., 2008). The results of other studies have shown that job mobility affects the areas of job satisfaction in different ways. External mobility increases satisfaction with objective working conditions and work-life balance, while internal mobility is crucial for satisfaction with future career prospects (Fasang et al., 2012).

Our study also showed that patient orientation plays an important role in job satisfaction. Simply, medical staff want to provide patients with high-quality care, and the joy and sense of fulfillment resulting from the provision of services affect job satisfaction (Harris et al., 2008). Also, previous research indicates that customer orientation positively relates to job satisfaction (Weisman and Nathanson, 1985; Nakata and Saylor, 1994; Leveck and Jones, 1996; Bateman and Organ, 2017; Todd Donavan et al., 2018). Healthcare professionals must respond to patients’ needs and enjoy serving those needs. Empathy for and responding to the patient’s needs are key elements of the medical profession. Research indicates that caring for patients is the most enjoyable aspect of the work of doctors and nurses and that empathy for patients and meeting their needs are important determinants of productivity and satisfaction (Albaugh, 2005). Many other studies have shown that the emotional nature of their patient-centered work and the associated need to control how emotions are expressed or suppressed affect the job satisfaction of healthcare professionals (Lu et al., 2005; Bartram et al., 2012; Gountas et al., 2014). These are important factors to consider as emotional labor and exhaustion from caring for a patient can affect healthcare workers’ jobs and overall life satisfaction (Demerouti et al., 2000). Therefore, a better understanding of how healthcare workers’ personal (psychological) resources contribute to job satisfaction should help healthcare facility managers make workflow decisions that benefit patients and healthcare professionals. Therefore, the tendency to empathize with patients and enjoy responding to their needs is important. As other researchers note, increasing salaries for medical personnel is not enough – medical personnel derive satisfaction from their internal motivations and desires (Cangelosi et al., 2008).

Among the demographic variables considered in this study were gender and the level of education of medical personnel. This study showed a significant effect of gender on job satisfaction (men reported higher job satisfaction than women). In other studies, this variable showed a significant relationship with satisfaction (Kavanaugh et al., 2006; Lu et al., 2016). For example, some researchers found that there is indeed an effect of gender on job satisfaction, in particular, satisfaction with pay and the work environment (Jung et al., 2007). According to studies using data from the United States, women reported higher job satisfaction than men and higher job satisfaction in female-dominated workplaces (Bender et al., 2005). In turn, a study conducted in China showed that gender, age, education, professional status, occupation and seniority significantly impacted doctors working in health clinics in cities and towns (Wei et al., 2005).

Our study also showed a significant impact of the stage of education on job satisfaction. This compound has also been described in other studies (Lu et al., 2016; Temesgen et al., 2018; Quyen et al., 2020). Higher education gives employees a better chance of choosing the right job and income, which translates into greater job satisfaction. People with higher education are more respected and hold higher positions. In addition, medical professionals with higher education in the workforce have more opportunities to participate in lifelong learning and capacity-building programs. This is an important element contributing to higher job satisfaction among medical personnel (Quyen et al., 2020). In studies conducted in China, healthcare professionals with the highest education were more satisfied with their independence and promotion opportunities, including a sense of professional achievement and co-workers. A high level of education helped them look for and get jobs with less stress (Rambur et al., 2005; Lu et al., 2016). Also, in the study of the relationship between job satisfaction and demographic characteristics in a sample of Turkish medical personnel, it was shown that education could be considered one of the factors affecting job satisfaction (Fikri et al., 2008).

According to the literature, strategies aimed at strengthening talent management practices should consider complementary actions regarding corporate social responsibilities (CSR), which relate to the efforts made by managers to ensure the well-being of various interest groups, including employees. Social responsibility toward employees includes various activities, such as communication and information flow, appropriate training, care for the health and well-being of employees, work-life balance and concern for safety in the workplace (Berber et al., 2014). According to the literature, appropriate CSR-oriented strategies allow entities to attract and retain the best talents, affecting TMP’s development and improving employee satisfaction and commitment (Santos-Jaén et al., 2021). CSR promotes a policy of flexible work, work-life balance, adequate earnings, development and remuneration in the workplace. All this increases employee motivation and satisfaction (Santos-Jaén et al., 2021). CSR practices can therefore play an important role in talent management, especially in the processes of attracting, motivating and retaining employees (Berber and Aleksic, 2016).

Proactive CSR motivates employees by offering training and development opportunities. CSR can be a powerful talent management tool by promoting positive attitudes, organizational culture and, as a result, job satisfaction among employees (Wang Y. et al., 2020; Gimeno-Arias et al., 2021). The literature on the subject indicated that CSR activities in a company focused on human resources management are perceived as positively impacting their attitudes and behaviors, which leads to employee satisfaction (Wang, 2018; Santana et al., 2020). So far, The results indicate that CSR practices are important for job satisfaction (Palacios-Manzano et al., 2021). Many managers agree that entities applying CSR practices more often evoke positive emotions in employees, who have greater identification with the organization and agree with its values (Onkila, 2015). An individual’s Responsible actions foster employees’ positive attitudes and behavior, including job satisfaction (Palacios-Manzano et al., 2021). CSR activities should increase job satisfaction because the demonstrated social responsiveness directly meets the employee’s social requirements toward the individual. Therefore, it can be argued that socially responsible units will take several actions that, by increasing CSR practices, improve job satisfaction among their employees (Berber et al., 2014; Palacios-Manzano et al., 2021).

Previous studies have shown that CSR activities positively impact human resource management by generating synergies and greater satisfaction among employees, which allows organizations to achieve their goals more effectively (Gimeno-Arias et al., 2021). However, these studies were based on results obtained only from a sample of Spanish companies. For this reason, these results cannot be extrapolated to other regions, as CSR depends on aspects such as culture, ethics, legislation and the economic environment. It would also be worth conducting them in relation to TMP in the healthcare sector. In subsequent studies, it would be worth checking whether TM practices do not play a mediating role between CSR and JS.

Although the results of this study highlight the role of talent management, social competence, patient orientation and job mobility in healthcare professional job satisfaction, limitations of the research should be noted. First, our analysis focused on four selected talent management practices that are believed to affect job satisfaction (i.e., talent motivation, professional development, employee appraisal, and organizational culture). Of course, other practices and other social factors (e.g., CSR) can also affect the overall job satisfaction of healthcare professionals. Therefore, future research that extends our framework is encouraged. Another study limitation was the lack of medical profession as control variables in the analysis. This survey lets you know the opinions of various health professionals, such as doctors, nurses, and dentists. As most other studies do, we do not limit our study to one occupational group, such as nurses or doctors. Still, we include all kinds of different health professionals. Future research should consider a study on talent management and its impact on job satisfaction in single medical occupations.

Little research has been done on the relationship between talent management and job satisfaction in healthcare professionals. So far, such studies have not been carried out in countries such as Poland, which is struggling with Europe’s greatest shortage of medical workers. The study deepens the understanding of how medical staff in Poland evaluate existing talent management practices in healthcare entities. This study also broadens the knowledge on the impact of talent management practices on job satisfaction in Poland’s healthcare entities, taking into account other organizational, behavioral and demographical factors (patient orientation, organizational culture, social competencies, gender, education stage).

## 5. Conclusion

Job satisfaction of medical personnel is a complex concept conditioned by many factors, the knowledge and identification of which may imply organizational changes necessary to improve the functioning of Polish public healthcare entities. One such factor is the quality of talent management. The study aimed to assess the quality of talent management in Polish healthcare entities and its impact on the job satisfaction of medical personnel. The study evaluated four talent management practices: talent development, talent motivation, employee appraisal and organizational culture. The listed talent management practices were shown to be underdeveloped in public healthcare institutions in Poland. The best-rated talent management practices were organizational culture and talent motivation, and the worst was talent development. This study confirmed that talent management has a strong, positive impact on job satisfaction. The impact of job mobility, social competence and patient orientation on job satisfaction is significant but lower. The demographic characteristics that significantly impacted job satisfaction were gender and education stage.

Public hospital management can use these results to manage talented healthcare professionals in order to increase their job satisfaction. This study shows that managers and decision-makers should adopt talent management practices in healthcare entities. The desired results, such as job satisfaction of medical staff, can be achieved by focusing especially on organizational culture and talent motivation. It is also necessary to carry out specific activities to improve the patient orientation, job mobility and education stage of the medical staff, e.g., staff education through workshops and training programs. We recommend that healthcare entities and health policymakers adopt new strategies and educational programs that include training as part of the curriculum for developing the social competencies supporting the patient orientation of medical staff. These approaches would result in the satisfaction of medical staff and patients and subsequently improve the quality of care.

## Data availability statement

The raw data supporting the conclusions of this article will be made available by the authors, without undue reservation.

## Ethics statement

The studies involving human participants were reviewed and approved by Warsaw University of Technology Senate Committee on Professional Ethics. Written informed consent for participation was not required for this study in accordance with the national legislation and the institutional requirements.

## Author contributions

WP: conceptualization, methodology, software, data collection, investigation, validation, writing—original draft, and writing—review and editing. MK-A: conceptualization, investigation, writing—review and editing, and supervision. All authors contributed to the article and approved the submitted version.

## Conflict of interest

The authors declare that the research was conducted in the absence of any commercial or financial relationships that could be construed as a potential conflict of interest.

## Publisher’s note

All claims expressed in this article are solely those of the authors and do not necessarily represent those of their affiliated organizations, or those of the publisher, the editors and the reviewers. Any product that may be evaluated in this article, or claim that may be made by its manufacturer, is not guaranteed or endorsed by the publisher.
